# Experiences of Stress – A Focus Group Interview Study Among Swedish Adolescents During the COVID-19 Pandemic

**DOI:** 10.1177/10598405211071002

**Published:** 2021-12-30

**Authors:** Moa Hörbo, Camilla Johansson, Tide Garnow, Pernilla Garmy, Eva-Lena Einberg

**Affiliations:** 1 Faculty of Health Sciences, 4342 Kristianstad University, Kristianstad, Sweden; 2 Department of Health Sciences, Faculty of Medicine, 5193 Lund University, Lund, Sweden; 3 Equal contribution as first author

**Keywords:** stress, experience, adolescents, COVID-19, focus group

## Abstract

Adolescence can be a stressful period in life. The period contains challenges associated with the transition from childhood to adulthood (body changes, changes in interpersonal relationships, and identity changes). The aim was to investigate experiences of stress among adolescents in addition to stress related to the COVID-19 pandemic. Focus group interviews (n = 8) were conducted with girls (n = 22) and boys (n = 19) aged 13–15 in southern Sweden. The transcribed interviews were analyzed with qualitative content analysis. Analysis of the collected material resulted in two categories with four sub-categories each of which highlights adolescents’ experiences of stress. The results show that adolescents’ have a variety of experiences of stress, i.e., what they mean are the sources of stress and how stress is manifested. The adolescents experienced how stress was manifested both physically and emotionally. This affected both their sleep and performance. The adolescents reflected on both positive and negative manifestations of stress.

## Introduction

Adolescence can be a stressful period in life. The classic definition of stress is a nonspecific response of the body to any demand for change (Selye, 1956). Survey studies have shown that adolescents feel stressed (Inchley et al., 2020). During 2020 and 2021, the world was thoroughly affected by the COVID-19 pandemic, and it has been a challenging period for adolescents worldwide, thus affecting stress and mental health (Branquinho et al., 2020; Donker et al., 2021; Guessoum et al., 2020; Nocentini et al., 2021; O’Sullivan et al., 2021). In Sweden, however, schools for children and adolescents aged 6–15 were mainly open during the pandemic in contrast to many other countries (Martinsson et al., 2021). It is therefore of interest to investigate the experience of stress among Swedish adolescents during the pandemic. A cross-sectional study with 1054 adolescents from Canada found that COVID-19 stress was related to loneliness and depression especially for adolescents who spend more time on social media (Ellis et al., 2020). Experience of stress among children in Sweden aged 10–12 had been investigated with focus group interviews before the pandemic (Warghoff et al., 2020); however, there is a gap in the literature regarding interviews about the experience of stress among adolescents aged 13–15 years as well as their experience of stress during the pandemic in Sweden. It is important to highlight adolescents’ experiences of stress to be able to provide good and adapted support.

## Background

Adolescence is a period of significant physical changes, changes in interpersonal relationships, and identity. Early adolescence extends between the ages 10–15, and middle adolescence between the ages 14–17 (WHO, 2010). From early adolescence, a concrete thinking (“here and now”) evolves into a more abstract thinking in the middle adolescence, however, this often goes back to concrete thinking under stress (WHO, 2010). Children approach adulthood and create their own identity during adolescence. According to Erikson's psychosocial development theory (Erikson, 1994), the individual goes through eight different phases during life. Each phase can be linked to a specific age range and involves a specific challenge that the individual needs to deal with. Since adolescence involves conflicting demands and an uncertainty for future, it can lead to a crisis in identity and role confusion. Emotional, physical, and social changes play a big part in adolescence and can be stressful life events (Erikson, 1994). The rapid and major changes that occur in the transition from childhood to adulthood can overload adolescents and make the problems challenging to deal with. This can lead to stress. Previous research has shown relationships between school-related stress and impaired mental health among adolescents (Hiltunen, 2017). A large study Health Behavior in School-aged Children (HBSC) with 220,000 children and adolescents aged 11–15 from 45 countries and regions in Europe and North America showed that mental illness among adolescents is increasing (Inchley et al., 2020).

Besides the developmental stressors, schoolwork also stresses adolescents. According to the HBSC-study, adolescents feel pressured by schoolwork, and this pressure increases with age (Inchley et al., 2020). Previous research has shown a relationship between low academic performance and negative emotions (Pekrun et al., 2017) as well as between school stress and impaired mental well-being (Hiltunen, 2017). Another type of stress is screen stress. The usage of smart phones tends to create altered sleep behavior and lead to experiences of stress. Screen stress affects adolescents’ sleep and impairs their ability to relax (Garmy & Ward, 2018; Hedin et al., 2020). It is thus important to listen to the voices of the adolescents about their stress experiences.

## Aim

The aim of this study was to investigate the experiences of stress among adolescents aged 13–15.

The specific aims were:

1. To investigate positive experiences of stress among adolescents.
2. To investigate negative experiences of stress among adolescents.
3. To investigate experiences of stress related to the COVID-19 pandemic.

## Methods

### Study Design

The study employed an inductive qualitative design (Polit & Beck, 2021) and was performed with focus group interviews analyzed with qualitative content analysis. The study was approved by the Regional Ethics Review Board in Lund, Sweden (EPN 2018/842). All procedures were conducted in accordance with the Declaration of Helsinki.

### Sample and Data Collection

Data collection occurred in August 2020 at one compulsory school in an urban area with 500 students aged 6–15 years. In Sweden, compulsory school includes public as well as private schools and attending school is mandatory from age 6–15. The school administration and the school nurse received information about the study and was asked to recruit students aged 13–15. Inclusion criteria were students aged 13–15 years. The school nurse distributed information letters and consent forms to the students and their legal guardians. After obtaining written informed consent from the students and their legal guardians, the school nurse divided the students into groups with an intention to create focus groups containing 4–8 participants in each group. All students who wanted to participate were included. The school nurse was also prepared for the participants’ possible reactions and need for support after the interviews. The first two interviews (the pilot interviews) only contained two participants in each focus group due to sick leave (n = 2) and declined participation (n = 2). The final sample consisted of 41 students (22 girls and 19 boys) distributed over eight different focus groups with 2–8 participants per group (Table 1). The sample size was determined on basis of previously conducted focus group studies with children and adolescents (Garmy et al., 2015; Hedin et al., 2020; Warghoff et al., 2020).

**Table 1.** Distribution of the Number of Participants, Gender, and Age in the Focus Group Interviews.

Focus group, number	Number of participants and gender	Age
1	1 girl, 1 boy	15 years
2	1 girl, 1 boy	15 years
3	4 girls, 3 boys	15 years
4	3 girls, 2 boys	13–14 years
5	6 girls	13–14 years
6	8 boys	14 years
7	3 girls, 3 boys	13–14 years
8	4 girls, 1 boy	13–14 years

The focus group interviews were conducted in the school during school hours. Before the interviews started, the participants were informed about the aim of the study, confidentiality, voluntary participation, and the right to decline participation at any time. The interviews were audio recorded. A semi-structured interview guide was developed by the fourth and fifth authors—both have PhD degrees and more than ten years of experience working as school nurses. The interview guide was reviewed by researchers with different competences: school nursing, public health, psychology, and pedagogy. The opening question was “What do you think of when you hear the word stress?” Other questions included “Can you please describe situations when stress was good”, “Can you please describe situations when stress was bad. The interview guide has been used in an earlier study in children aged 10–12 (Warghoff et al., 2020). Although the age group was different, the interview guide worked well.

Adjustments were made regarding the ongoing COVID-19 pandemic. The earlier study with children aged 10–12 was conducted in 2019, i.e., before the pandemic. In the current study, we therefore added a question about if the pandemic affected the students’ sense of stress. The first two authors, registered nurses and master's students, participated in all focus group interviews and took turns being the moderator and the observer. The moderator's role was to conduct the interview, and the observer provided follow-up questions at the end of the interview (Wong, 2008). During the two initial pilot interviews, the fourth author—a pediatric nurse with extensive experience as a school nurse and conducting focus group interviews—participated and acted as a moderator in the first interview and observer in the second. The third author is a psychiatric nurse, and the last author is a pediatric nurse with extensive experience as a school nurse. None of the interviewers and authors knew the adolescents before or worked at the school where the data were collected. The interview guide was evaluated after the initial pilot interviews and was found to be adequate. The pilot interviews were successful and were therefore included in the analysis.

### Data Analysis

The focus group interviews were transcribed verbatim by the first two authors. The material was then analyzed with a qualitative content analysis (Graneheim & Lundman, 2004). The first two authors started to read the material several times and then identified meaning units and then condensed and coded the text. The last author was then invited to work together with the first two authors to group the codes. The analysis went back and forth, and the material was finally divided into eight sub-categories. Two categories were thus identified. At the end of the analysis process, all authors discussed the results until consensus was achieved.

## Results

The analysis of the collected material resulted in two categories with four sub-categories each of which highlights adolescents’ experiences of stress (Table 2). The results show that adolescents’ have a variety of experiences of stress, i.e., what they mean are the sources of stress and how stress is manifested.

**Table 2.** Adolescent Experience of Stress.

Sources of stress	Manifestation of stress
Prioritizing between school and leisure time	Increase and decrease of performance
Demands and conflicts	Sleep problems
Fear of missing out	Physical reactions
Worries about the pandemic	Emotional reactions

### Sources of Stress

The adolescents experienced different sources of stress: difficulties in prioritization between school and leisure, demands and pressure due to relationships, fear of missing out, and worries about the ongoing pandemic.

#### Prioritizing between school and leisure time

The adolescents experienced difficulties in prioritizing between schoolwork and leisure activities, which caused stress. Some chose to prioritize schoolwork over other activities while others found that their activities took time from schoolwork. The adolescents reported having difficulties planning, prioritizing, and controlling school and leisure time activities. They felt high pressure on themselves since the demands for performing well were high in both school and leisure time activities. No matter what they did, they felt bad because they had to prioritize something away. Many found that it was too much at the same time and that everything collided. The stress related to this made them have a constant feeling of not managing, which sometimes led to a feeling of wanting to give up school:

*Then, for example, at night from nowhere you start to think it's a test in three days and you do not know what to train and from nowhere you just start to cry, and above that a lot of assignments and many tests at once. It is just too much! It makes me just skip school. Girl, Focus group 4*.

*Yes, when you exercise quite a lot, you do not have much time for school and then it can be stressful. Girl, focus group 8*.

The students could also feel fear of missing out in school because, for instance, sick leave could lead to school failure. During the COVID-19 pandemic, students were not allowed to enter school even with minor symptoms. School failure was especially stressful because it could possibly have an impact on the adolescents’ entire future:

*When you kind of feel like you’re not keeping up, then I can get stressed. Boy, focus group 7*

#### Demands and conflicts

Although friends and family mainly were a positive force and minimized stress, the adolescents also experienced how stressful and complicated relationships could be. The adolescents experienced those relationships with parents and peers and found that they could cause stress due to different perceived demands and conflicts. When the parents had high demands on school performance it caused stress because the adolescents did not want to disappoint them. They felt that their parents were stressed over school results, which made the adolescent stressed as well. At the same time, the adolescents found that they were required to help out at home, e.g., tidying up their room or hanging laundry while studying. This increased the stress:

*Then I can also get stressed because now I have to do this so that they [my parents] are not disappointed in me now, it is also a kind of stress. Girl, focus group 3*

*I kind of get more careful like this, if you do something, they can get stressed by it and they [my parents] get a little angry at me. Girl, focus group 1*

Parents’ stress overall had an impact on the adolescents’ experiences of stress especially when the adolescents felt that they caused their parents’ stress. On the other hand, the adolescents experienced a decrease in stress when parents made reasonable demands. Peer relations could also create stress due to arguments or conflicts. The fear of not being able to resolve the conflict caused stress; bullying could also create stress and fears of going to school. The adolescents also described stressful situations at home when parents were divorcing:

*My parents were divorcing this summer, then it was very hard. I could not breathe because all my crying. I cannot describe, I had never cried so much before. It came from nowhere. I really could not breathe. I had to be quiet because I didn't want my parents to hear me from my room. I did not want them to hear that I was sad; it was really hard. Girl, focus group 5*

#### Fear of missing out

The smart phone was constantly present in the adolescents’ lives. Being constantly connected, participating, and communicating on social media was experienced as fun, but it could also be stressful. The fear of missing out was stressful when the flow of notices and messages was constantly ongoing:

*My whole phone stresses me, when I sit and study, my hand is pulled to pick up the phone and go on social media. It stresses me that way. Girl, focus group 5*

Beauty ideal on social media also created stress especially among the girls:

*Everyone wants to be slim and perfect and if you are not then you feel … I do not know… sometimes I can just feel kind of bad because I do not look like that. Girl, focus group 2*

Others did not experience stress in social media specifically but felt that the smart phone overall caused stress. The constant connectedness had a negative impact on school performance. The adolescents found that the phone made it difficult to focus on the schoolwork because they were constantly interrupted. Some had to uninstall apps to get schoolwork done but felt an immediate need to reinstall it again for fear of missing out.

#### Worries about the pandemic

The adolescents had different experiences of stress related to worries about the ongoing pandemic. In the conversations, it emerged that some of the adolescents had felt stress and anxiety at the beginning of the pandemic; others were not affected at all because they felt that they could live on as usual. Some described that their leisure time activities had been canceled. Also, descriptions of worries and stress appeared due to the uncertainty for the future:

*I’m a little stressed about how it will be or a little worried about how it will be in the future. Will you be able to go abroad in the same way? Will you be able to hug your grandmother and grandfather as long as you want? Girl, focus group 8*

*I have not been so much affected by it [the pandemic] but you do not know what will happen. Boy, focus group 2*

*This is what you have become worried about: Imagine if someone in the family would get sick. Girl, focus group 5*

### Manifestation of Stress

The adolescents experienced how stress was manifested both physically and emotionally, and it affected both their sleep and performance. The adolescents reflected on both positive and negative manifestations of stress.

#### Increase and decrease of performance

Many of the adolescents viewed stress as something negative, but some felt that it could also have a positive impact on performance. In these cases, stress could be a driving force—a performance enhancer. It could lead to increased motivation and focus both on schoolwork and leisure time activities:

*I could not have been without the stress; I had not done anything. Boy, focus group 3*

The adolescents felt at their best when there was no reason to stress, but at the same time they felt that a life entirely without stress would make them do nothing at all. A fair amount of stress made the adolescents feel that the task was manageable. Too much stress, on the other hand, made it difficult to focus and decreased motivation. They reported that stress led to experiences of not having enough time to do what they needed to do:

*You can have it on a certain level … you really need stress to get to grips with things. Then there is this annoying stress that you would rather avoid. Girl, Focus Group 5*

Stress was sometimes used as a warning signal that made the adolescents stop if it became too much.

*For me, stress can have a bad effect because it becomes stressful because then you cannot think clearly either; thus, even if you work with a certain thing, it may not be good anyway even though you have to do it in time because then you think of all the others things you have to do too. Boy, focus group 2*

*So, I get kind of in a bad mood—I get angry and then with many homework then I perform worse on each homework instead of doing one at a time. Girl, focus group 2*

#### Sleep problems

The adolescents reported that stress had a negative impact on their sleep. They had difficulties in letting go of stressful thoughts that affected sleep and sometimes led to sleeplessness. Also, schoolwork could make them stay up late at night—sometimes the whole night. This in turn caused more stress because they knew that they did not get the recovery that they needed:

*If I have stress, then it becomes harder to sleep. Then I only think about what I am stressed about. Girl, Focus group 2*

*When you are stressed, you just want to sleep. Boy, focus group 6*

*Yes, stress can affect [sleep] especially if it is in the evening or afternoon so you still have to keep it at night—either stay up during the night and think about it or manage to fall asleep but you cannot sleep so well anyway; thus, the head is still working with this so then it can continue all night and you can still keep it in the morning. Then it will be like this and I do not want to go to school. Stress can affect in that way. Boy, Focus group 2*

#### Physical reactions

The adolescents experienced physical reactions of stress such as a lump in the throat, chest pain, headache and stomachache. This could even make their own stress levels increase:

*During a conference when I had to talk, I was very stressed and had a stomachache. I had to pretend like nothing when I got up. Then I did not know how it would be. Girl, focus group 7*.

*I kind of get a lump in my throat [when I’m stressed]. Girl, focus group 8*

The adolescents reported that people who are stressed can get more tired than they usually are, but they paradoxically also feel turned up with more energy. Sometimes they could identify people around them as being stressed just by looking at their body language:

*[You notice that someone is stressed by] you kind of feel that they are stiff and so on.. in the body language and so on. They are not so calm and they are kind of high on guard and stuff. Boy, focus group 1*

#### Emotional reactions

Stress could cause emotional reactions in the adolescents. They could experience irritability, panic, nervousness, sadness, disappointment, and also aggressiveness:

*[When I am stressed] I feel anger. Boy, focus group 8*

*I get angry at anything [when I am stressed]. If someone talks to me, I just get really angry. Boy, focus group 4*

Sometimes they could reflect on their reactions as being too powerful, but the stress could also make them “turn off” and not want to talk to anybody. The adolescents said that what peers say and think can hurt mentally. The students reported how they were thinking back and wanted to change things, but it was already too late. The adolescents also reported how they could get anxious, sad, and lonely when they are stressed:

*When you are stressed, you can get a little anxiety and stuff… you are sad and feel lonely and stuff. Girl, focus group 3*

Emotions of anger and sadness were experienced when the adolescents found out that they did not have the time for doing things that were important for them. The adolescents also gave examples of being stressed: They did not want to talk very much. They had a feeling of being down. Other students reported that they became more easily irritated when they were stressed. They could take it out on other people instead of dealing with oneself. Students also spoke of being nervous and annoyed when they were stressed.

## Discussion

The current study started in the middle of the ongoing COVID-19 pandemic. During the data collection in August 2020 there was low spread of infection with relatively few restrictions in Sweden. A study by Kapetanovic et al. (2021) revealed that adolescents felt increasingly bad during the pandemic. The restrictions have affected adolescents’ everyday lives and relationships according to a cross sectional study from Canada (Ellis et al., 2020). The adolescents felt isolated and lonely and experienced more conflicts at home with their parents (Ellis et al., 2020). Furthermore, the increased stress associated with distance education was described because the same clear routines no longer existed (Kapetanovic et al., 2021). In the current study from Sweden however, most adolescents stated that they were relatively unaffected by the pandemic. Some of the adolescents felt anxious and stressed that someone close to them would become ill. They worried about older relatives who were part of risk groups and had thoughts about how traditions, festivals, and opportunities to travel would affect the adolescents later on. A recent study with focus group interviews with school nurses in Sweden during the pandemic found that the school nurses experienced a transition to a digital way of working. However, the school nurses still found that they were available and could provide adequate support to the students (Martinsson et al., 2021).

Difficulties prioritizing between school and leisure time was stressful according to the adolescents. They also reported that school and homework took a lot of time from their free time and this contributed to increased stress. Several of the adolescents described difficulties in planning and prioritizing. Girls often opted out of their free time and focused on schoolwork because there was a fear of not being able to perform well in school. Many boys prioritize leisure time including sports. Whatever they planned, their priorities caused stress due to a guilty conscience. Similar results were seen in a study by Wilhsson et al. (2017) where adolescents did not have the ability to prioritize and plan their everyday lives, i.e., how they should allocate time for school and leisure. This could also be about the adolescents not being able to reflect on their time, which could be explained by their pressured situation. This contributed to the adolescents feeling that they were forced to prioritize either schoolwork or leisure activities in their struggle to balance their time. The adolescent's situation manifested itself in stress-related symptoms and could be part of their difficulties in prioritizing and planning their everyday lives. This dilemma could be a topic during health conversations with the school nurse. Students appreciate when they can take part and be empowered to talk about issues that they find important and not simply stick to the school nurse's pre-defined agenda (Golsäter et al., 2011). With knowledge from this study, school nurses can be open to pick up such topics when relevant. A dissertation by Hiltunen (2017) reported that students’ consciences were affected when leisure time was prioritized. According to Wilhsson et al. (2017) adolescents need support to deal with the school-related stress they experience. Stress can be reduced by supporting adolescent's ability to plan and visualize how time is used and to strengthen their belief in their own abilities. Stress can also be reduced by developing the dialog between school and home (Wilhsson et al., 2017). The school nurse is a significant resource in this work. The school nurse can promote health and wellbeing through health conversations by allowing the student to reflect based on their own situation and their own conditions (Golsäter et al., 2011).

The adolescent experiences high demands on performance in school and at home. The results showed that the adolescent experienced parents having high demands on their performance. They reported that they wanted to perform well in school and at the same time provide support at home. This made the adolescents place high demands on themselves so as not to disappoint their parents. Warghoff et al. (2020) conducted a focus group study among Swedish children aged 10–12 and they found that the children want good grades so that the parents would not be disappointed. According to Hjern et al. (2008), adolescent's demands related to school increased between the ages of 13 and 18. This in turn caused school stress which in turn caused psychological problems. Hiltunen (2017) found a pattern linked to adolescent's school performance where stress-related ill health was seen as a result of an imbalance between internal and external demands. Furthermore, a requirements dimension was described that concerned how the adolescent approached their schoolwork in relation to requirements. This can come from both inside and outside. High internal and external demands are perceived as a significant strain, which results in stress reactions in the body (Hiltunen, 2017). It is important to balance demands and support from the environment to maintain one's health (Wiklund et al., 2012). A prior study (Eriksson & Sellström, 2010) showed that the risk of ill health was about 50% higher in classes with high demands versus reasonable demands. Furthermore, the results showed that there was a difference between girls and boys: Girls had higher demands on themselves in the school class (Eriksson & Sellström, 2010). The challenge is to find a balance between very high demands and very low demands and to have realistic demands. School health staff and teachers play a crucial role in creating a favorable school climate. Therefore, efforts aimed at supporting teachers to set realistic demands and expectations can improve the school climate. Erikson's development theory (Erikson, 1994) reported that a lack of help and encouragement as well as very high demands can cause feelings of inferiority. High demands on adolescent's performance and the accompanying feelings of not being able to live up to these expectations can cause the adolescent to feel inadequate. School nurses have the opportunity to impact students via health dialogs (Garmy et al., 2021) including conversations about mental health (Jönsson et al., 2019), to identify and support students experiencing stress-related problem, and refer students further if needed. School nurses may also work with students at the group level and or together with other school personal, e.g., the school social worker at school (Martinsson et al., 2021) can prevent stress or promote health.

The adolescents have an ambivalent attitude to the smart phone's impact on stress. The adolescents seen here spent a large part of the day using their smart phone. They described smart phone use as something positive with the social interaction with friends, but that the constant connection at the same time created stress. A recent study (Hedin et al., 2020) found that adolescents described their smart phone use as addictive. Several of the adolescent described that they had difficulty sleeping if they used social media before going to bed. The adolescent had different views on the parents’ commitment. Some of the adolescent said that their parents had no idea about their smart phone use, activities on social media, and how often they engaged in it during the night. Some of the adolescents mentioned that they wished their parents had more rules about media use, which would make it easier to put away the smartphone and they would not have to make the decision themselves because since it would help them feel relieved (Hedin et al., 2020). Similar results were seen elsewhere (Garmy & Ward, 2018) where adolescent who used the smart phone and social media before bedtime and those who slept with the smart phone in the same room had difficulty relaxing. If they turned off the cell phone, then they felt anxious and stressed that they were missing something. This also resulted in increased fatigue during the days. Another study (Hiniker et al., 2016) examined how families set up rules for smart phone use. The results showed that it was easier for the adults to ban or restrict various activities on the smart phone, e.g., to ban the use of Snapchat than to ban the use of the smart phone completely. The results also showed that parents and children agreed that parents should also relax when spending time with the family (Hiniker et al., 2016). The findings from our study showed the mixed feelings concerning smart phones as both relaxing and stressful.

Erikson's development theory (Erikson, 1994) found that adolescents during this period seek out different social groups with different norms and values. The youth usually change groups several times and embrace the new group's ideals and values with equal enthusiasm each time. Uncertainty about which ideals and values one should orient oneself to can increase sharply if the surrounding society is in a time of rapid transformation. During this developmental phase, peers have an important role for the individual's development of identity and self-esteem (Erikson, 1994). The opportunities today for more social contacts and groups with the help of social media mean that adolescent have more opportunities to find their identity. A challenge is that many adolescents see the smart phone and social media as an extended arm of themselves and their identity. This can make it difficult to limit the use. Phone use is also a relevant topic for health conversations with the school nurse.

## Strengths and Limitations

The strengths of the current study include the large sample size that allowed different voices to be heard. The goal was to conduct focus group interviews with 4–8 students in each group. However, only two students turned up in two focus group interviews. Both interviews with only two students were rich and were thus included in the data analysis. A limitation is that all students came from the same school, and therefore transferability must be considered. Sweden did not lock down during the COVID-19 pandemic as many other countries did. The schools for students aged 6–15 were open almost all the time, and thus the students came to school and only used distance education occasionally during the pandemic; therefore, it might not be possible to transfer the findings from this study to other countries. However, the experience of stress in adolescents is prevalent regardless of an ongoing pandemic or not. Worries regarding the pandemic, conflicts, difficulties in prioritizing between school and leisure would likely be seen in other contexts.

## Conclusions

Adolescents’ have different experiences of stress, i.e., how they define the sources of stress and how stress is manifested. The adolescents reflected on both positive and negative manifestations of stress.

## Implications for School Nursing and Future Research

The results of the current study shed light on experience of stress that affect adolescent's health and wellbeing. The school nurse can, through health conversations, introduce knowledge about stress and the effect of stress on the body. School nurses can identify signs of stress and mental health issues among students with different needs (Berglund Melendez et al., 2020; Jönsson et al., 2019; Musliu et al., 2019) and are important in the work to support a positive mental health development among adolescents (Garmy et al., 2014). School nurses can also work in teams with other school health professionals such as the school social worker to address issues of stress among adolescents both on an individual and group level. The school nurse can contribute to sustainable development by preventing mental illness and psychosomatic symptoms by working to promote health.

Further research is needed to get the perspective of parents and school nurses of stress in adolescents as well as their experiences of opportunities to provide support. Using both survey and interviews may give complementary knowledge about the topic. Moreover, interventions could be developed within school nursing practice to support stress-management aimed at adolescents and their parents.
